# ADAMTS13, TTP and Beyond

**DOI:** 10.4172/2161-1041.1000e104

**Published:** 2012-09-03

**Authors:** X Long Zheng

**Affiliations:** Department of Pathology and Laboratory Medicine, The Children’s Hospital of Philadelphia and The University of Pennsylvania Perelman School of Medicine, Philadelphia, PA 19194, USA

**Keywords:** Von willebrand factor cleaving protease, ADAMTS, Thrombotic thrombocytopenic purpura, Inflammation, Thrombosis, Atherosclerosis

## Description

ADAMTS13 (A Disintegrin And Metalloprotease with ThromboSpondin Type 1 repeats, 13), a plasma metalloprotease, cleaves von Willebrand factor (VWF) [1,2]. This cleavage is crucial for reducing the size of VWF multimers and adhesiveness, thereby preventing excessive platelet aggregation and thrombus formation at sites of vascular injury [3,4]. Deficiency of plasma ADAMTS13 activity could result in an accumulation of newly released ultra large (UL) VWF on endothelial cells where it is synthesized [5] and in blood [6], leading to a potentially fatal syndrome, thrombotic thrombocytopenic purpura (TTP).

TTP is clinically characterized by profound thrombocytopenia and microangiopathic hemolytic anemia (low hematocrit, elevated serum lactate dehydrogenase, and fragmentation of red blood cells) [7]. Some patients may present signs and symptom of neurologic and/or renal abnormalities [7]. There are two major types of TTP: Hereditary and acquired. Hereditary TTP is caused by inherited mutations of *ADAMTS13* gene, which result in severe deficiency (<5%) of plasma ADAMTS13 activity [1]. Acquired TTP, on the other hand, is primarily caused by acquired immunoglobulin G (IgG) autoantibodies against ADAMTS1 [8,9]. The autoantibodies bind the exosites that are critical for substrate recognition, thereby inhibiting the cleavage of VWF by ADAMTS13. Recent studies have shown that the spacer domain, particularly those solvent exposed residues comprising exosite 3, appears to be frequently targeted by the IgG autoantibodies in patients with acquired TTP [9–12]. A subtle modification of this exosite creates ADAMTS13 variants that retain proteolytic activity, but exhibit significantly reduced binding and inhibition by a panel of patients’ autoantibodies [10].

TTP must be distinguished from other conditions that cause thrombotic microangiopathic findings, which may be ADAMTS13 independent. These conditions include thrombotic microangiopathy occurring after hematopoietic progenitor transplantation [13], disseminated malignancies [14], certain drugs or chemotherapies [15], and infections [16], etc. The data available to date demonstrate that the assay of plasma ADAMTS13 activity and inhibitory autoantibodies may helpful in differential diagnosis, stratification of patients for a more targeted therapy, and prediction of relapses/prognosis [17]. A number of tests have been developed and validated for clinical use. An assay based on fluorescence energy resonance transfer (FRET) and a peptide containing 73 amino acid residues from the central A2 domain of VWF (FRET-VWF73) [18] has gained its popularity due to its simplicity and rapid turn around time. However, an interpretation of this test results should be cautious and a repeated test may be necessary in some cases. In addition, the correlation between the result of FRET assay and that of multimeric assay is relatively poor [19], suggesting the complexity in assessing plasma ADAMTS13 activity in patients.

Plasma infusion or exchange remains the mainstay of treatment for patients with TTP. It reduces mortality rate to less than 20% (7;20). For hereditary cases, an intermittent infusion of 2 units of fresh frozen plasma (FFP) every 2–3 weeks appears to be sufficient to raise plasma trough levels of ADAMTS13 activity to 0.05–0.1 unit/ml, which prevents the recurrence of acute burst [20]. However, life-long treatment with FFP remains inconvenient and the risk associated with the use of plasma product remains a concern, particularly in pediatric population. Gene therapy with self-inactivated lentiviral vector or an adeno associated viral vector (AAV)-mediated expression of a full-length or a carboxyl-terminal truncated form of ADAMTS13 (MDTCS) for the correction of hereditary TTP has been evaluated in a murine model of TTP. These results are encouraging [21]. For acquired TTP, plasma exchange (1.5x plasma volume, daily) is often required to replenish the missing ADAMTS13 protease and to remove autoantibodies that inhibit plasma ADAMTS13 activity [22]. Plasma exchange may be discontinued after achieving complete resolution of neurologic symptoms and thrombocytopenia. Approximately 30% of TTP patients may experience relapse, plasma exchange therapy should be reinstalled as early as possible. In addition, these patients may be benefited from additional adjunctive therapies including corticosteroids, cyclosporine and/or cyclophosphamide, and anti-CD20 monoclonal antibody (rituximab), etc. [20,23]. Novel therapeutics for hereditary or acquired TTP has been evaluated in animal models or clinical trials with some success. These novel modalities include the use of anti-platelet glycoprotein 1b (GP1b) monoclonal antibody, nanobody to GP1b, anti-VWF A1 aptamer, and N-acetylcysteine [24]. All these strategies involve in a direct disruption of the interaction between VWF and platelets, which is the pathologic hallmark of TTP and other related arterial thrombotic disorders.

Besides causing TTP, deficiency of plasma ADAMTS13 activity and/or increase of plasma VWF concentrations have been shown to be the risk factors for myocardial infarction [25], ischemic cerebral infarction [26], preeclampsia [27,28], and cerebral malaria [29]. These epidemiologic findings are consistent with the results obtained from animal studies. For instance, mice with a genetic ablation of Adamts13 gene (Adamts13^−/−^) exhibit a greater propensity for post ischemic perfusion injury to the heart and the brain than wild type mice or Adamts13^−/−^ mice receiving an intravenous infusion of recombinant human ADAMTS13 prior to the injury [30,31]. Moreover, Adamts13^−/−^ mice develop more and larger atherosclerotic plagues in the aorta and aortic arches than wild type mice after fed normal chew or high fat diet [32,33]. Image analyses by intravital microscopy and immunohistochemistry have revealed that leukocytes adhered to the injured vessel walls or monocytes infiltrated into the aortic tissues i.e. macrophages are dramatically increased in Adamts13^−/−^ mice compared with those in wild type mice [32,33]. These results indicate that ADAMTS13 plays a role in attenuating acute and chronic inflammation, thereby preventing ischemic/perfusion injury in the heart and brain, as well as the development of atherosclerosis.

In conclusion, the discovery of ADAMTS13 from study of patients with a rare form of hematologic disease, TTP, has provided us with an invaluable tool for further understanding the mechanism of hemostasis and thrombosis and many other related disease processes. Therefore, a success in the development of a novel therapy for TTP may be applicable to the therapeutic intervention of other related diseases due to the abnormality of the ADAMTS13/VWF axis.
